# Anesthetic Management of a Patient with Sustained Severe Metabolic Alkalosis and Electrolyte Abnormalities Caused by Ingestion of Baking Soda

**DOI:** 10.1155/2014/930153

**Published:** 2014-08-10

**Authors:** Jose Soliz, Jeffrey Lim, Gang Zheng

**Affiliations:** Department of Anesthesiology and Perioperative Medicine, MD Anderson Cancer Center, Houston, TX 77030, USA

## Abstract

The use of alternative medicine is prevalent worldwide. However, its effect on intraoperative anesthetic care is underreported. We report the anesthetic management of a patient who underwent an extensive head and neck cancer surgery and presented with a severe intraoperative metabolic alkalosis from the long term ingestion of baking soda and other herbal remedies.

## 1. Introduction

Little is known about the impact of alternative medicine on perioperative anesthetic care. We present a case of a patient who was under the long term management of a naturalistic doctor for a history of colon cancer. His prescribed treatment remedy constituted of a combination of herbs and alkaline fluid. This history was not elicited preoperatively. His intraoperative arterial blood gas test revealed unexpected profound metabolic alkalosis and multiple electrolytes abnormalities. This case presented some unique challenges in intraoperative hemodynamic and electrolyte management.

## 2. Case Report

A 63-year-old 75 kg male with a history of sinonasal melanoma presented for an endoscopic ethmoidectomy, sphenoidectomy, maxillectomy, palatectomy, and bilateral neck dissection. His past medical history included a 12-year history of colon cancer, which had been managed with a “nature remedy” in Mexico. His remedy included 4Life Transfer Factor Plus, Choice Fifty, Orasal, Boluoke, Grano Derma, Resveratrol Plus, and vitamins C, E, and D. He denied any other medical or surgical issues in the past. His laboratory report 14 days prior to surgery revealed potassium 3.2 mEq/L, chloride 94 mEq/L, and bicarbonate 31 mEq/L. All other electrolyte, renal, and liver function labs were within normal range. On preoperative anesthesia assessment, his vital signs were within normal limits, and his physical exam was normal with the exception of a large mass on the right side of his face.

He was brought to operating room with 2 mg of midazolam premedication. Upon arrival at the operating room he became difficult to arouse. General anesthesia was induced with 150 mg of propofol, 20 mcg of sufentanil, and 70 mg of rocuronium and was maintained with air, oxygen, and 0.6-0.7 MAC of desflurane plus 0.1 mcg/kg/hr of sufentanil infusion. After 1.5 hours of surgery, the patient received one liter of plasmalyte with minimal blood loss. A baseline arterial blood gas reported severe metabolic alkalosis and multiple electrolytes derangements (Table 1 at 0919). A blood chemistry test confirmed the accuracy of blood gas report. Therefore, 1 gram of calcium chloride, 2 grams of magnesium sulfate, 40 mEq of potassium chloride in 800 mL of normal saline (an institutional policy for peripheral IV potassium), and 100 mg of hydrocortisone were given. The ventilator settings were adjusted to target the ETCO_2_ of 40 mmHg. After about 2.5 hours into the surgery, his blood pressure decreased from 110s/70s mmHg to 80s/60s mmHg. Meantime, he had a total fluid balance of 4,000 mL of plasmalyte, 700 mL of blood loss, and 175 mL of urine output. After several boluses of vasopressin and phenylephrine, a 4-unit/hr vasopressin infusion was added to his treatment. His BP remained low in 80s/60s mmHg, for which he frequently required boluses of phenylephrine and ephedrine. His EKG showed regular sinus rhythm with rate of low 80s bpm. The total anesthetic duration was 12 hours. At the end of surgery, he received a total of 7600 mL of plasmalyte, 1100 mL of colloid, and 797 mL of packed blood cells, with 1000 mL of estimated blood loss and 595 mL of urine output. In addition, he also received second dose of 1 gram of calcium at the 7th hour and 40 mEq potassium at the 9th hour of surgery. Table 1 illustrates his intraoperative arterial blood gas results at different time points. He was extubated at the end of surgery in the operating room.

Postoperatively, he developed hallucinations described as “constantly dreaming and difficulty to close eyes.” The hallucination disappeared on postoperative day 3 without intervention. His K, Na, and CL remained low until postoperative day 6. His postoperative EKG showed a normal sinus rhythm with a rate of 70s bpm. No other issues were identified in his postoperative course. The patient was discharged to home on postoperative day 7.

On postoperative followup, we learned that, for the past 12 years, the patient used herbal medications and also drank an “alkaline liquid”: a mixture of 1 liter of pH 7.8–8 water, typically with bottled water, key lime juice, and a quarter of tablespoon baking soda. The endpoint of the remedy was to achieve urine pH of 7.58–8. He stopped all the herbs 5 days prior to surgery per his doctor's advice but kept his “alkaline liquid” until the night before surgery. He never experienced muscular cramping, weakness, or alter mental status in the past.

## 3. Discussion

Baking soda (sodium bicarbonate) has been widely used by people for reasons ranging from increasing excise tolerance to treating acid reflux disease and other medical conditions. Severe cardiac dysrhythmia and death after ingestion of sodium bicarbonate have been reported [1–4]. Ingestion of baking soda results in multiple issues including metabolic and electrolytes derangement. Details on pathophysiological influences of metabolic alkalosis and electrolytes abnormalities are beyond the scope of a case report.

Generally, treating severe metabolic and electrolyte abnormalities during anesthesia is based on patient's baseline condition and clinical symptoms. In our case, the lack of knowledge of etiology and baseline values led to difficulty in determining the endpoint of electrolyte correction. Our goal for this case was to partially restore potassium to a range of 3–3.5 mEq/L in light of his previous potassium 3.2 mEq/L to minimize the risk of intraoperative cardiac dysrhythmia. Without knowing his baseline sodium value we decided to use plasmalyte rather than hypertonic saline to avoid the risk of acute sodium overcorrection from his baseline level. This case presented a couple of unique issues. First, the patient developed refractory hypotension despite fluid replacement with the volume that seemed adequate in other similar cases. This might reflect the combined effects of hypovolemia and direct vasodilation from bicarbonate. Kaplan et al. found in their study that bicarbonate is a direct peripheral vasodilator. Under general anesthesia with halothane it caused profound hypotension in health volunteers [5]. Another study showed that even mild metabolic alkalosis induced by bicarbonate resulted in symptomatic hypotension during renal dialysis and required a larger volume infusion than in non-alkalotic patients [6]. Attenuated response to vasopressors was also observed in this case. Despite multivasopressor therapy in addition to fluid replacement, we were not able to achieve sustained blood pressure close to his baseline level. This might have reflected that, under a state of severe alkalosis, the response to vasoactive agents is blunted. Indeed, combined effects of left shift hemoglobin dissociation curve with tissue hypoxia caused by alkalosis, a low perfusion status, and multielectrolyte derangement had put the patient in a high risk of an ischemic event.

Secondly, we observed that the patient presented with a decreased response to stimuli. As described above, with a low anesthetic level, without extra adjustment of anesthetics the patient did not show HR and BP changes in response to multiple skin incisions and head repositions. We suspect that the decrease in anesthetic demand was secondary to a decrease in central nervous system (CNS) response to stimuli secondary to the CSF alkalosis. Sustained metabolic alkalosis with significant multiple electrolyte derangements under general anesthesia is a rare clinical scenario. Little is known about the potential complications and drug pharmacodynamics and pharmacokinetics under this situation.

It is a very common and misleading opinion that alternative medicines are benign and safe, as these remedies have been used worldwide. Lack of regulation and standardization of alternative medicine has made clinical evaluation of patients and their remedies challenging. More importantly, not knowing the impacts of alternative medicine to perioperative patient care will significantly undermine the perioperative risk stratification. Hence, developing an interview protocol and actively seeking history of alternative remedies rather than “patient to tell” during preoperative interview should be as important as the retrieval of any other medical and surgical information.

## Figures and Tables

**Table 1:** Intraoperative laboratory values.

	Time					
	0919	0954	1115	1226	1435	1738
pH	7.65	7.53	7.48	7.49	7.51	7.44
CO_2_	35	44	47	44	41	46
O_2_	198	193	183	205	202	266
HCO_3_	39	37	35	34	33	31
Base excess	17	13	10	9	9	6
Sodium	123	124	127	126	127	129
Potassium	2.6	2.5	3.3	3.4	2.9	3.2
Glucose	157	208	230	258	271	225
Calcium (ionized)	0.89	1.16	1.18	1.03	0.98	1.11
Lactate	1.3	1.8	1.9	2.3	3.1	3.5
Hematocrit	42	38	31	26	34	31
